# Planar ICP Assisted One-Step Synthesis of pH-Responsive PDEAEMA Polymer Thin Films

**DOI:** 10.3390/polym18030421

**Published:** 2026-02-06

**Authors:** Zahide Tosun

**Affiliations:** Department of Physics, Selcuk University, Konya 42075, Türkiye; zahidetsn@selcuk.edu.tr

**Keywords:** plasma polymerization, pH-responsive polymer, PDEAEMA, PECVD, functional coating, responsive surfaces, thin film, plasma diagnostics

## Abstract

Smart polymers have attracted significant scientific interest in recent years because of their capability to modify their physical and/or chemical properties in response to external stimuli, including temperature, pH, and electric or magnetic fields. Poly(2-(diethylamino)ethyl methacrylate) (PDEAEMA) is a pH-responsive polymer with significant potential for biomedical applications. Significant research has focused on the synthesis of PDEAEMA polymers given their potential in smart polymer applications. In this study, PDEAEMA thin films were synthesized via a planar inductively coupled plasma (ICP) system at 13.56 MHz in both continuous and pulsed modes. The effects of substrate temperature, plasma power, and plasma pulse off time on polymer surfaces were systematically studied. The deposited polymer films were analyzed for their chemical composition and structural properties using X-ray Photoelectron Spectroscopy (XPS) and Fourier Transform Infrared Spectroscopy (FTIR). Additionally, the plasma environment was analyzed using Optical Emission Spectroscopy (OES). Results indicated that polymers prepared under pulsed plasma conditions more closely retained the structure of the monomer. Moreover, the deposition rate increased as the plasma pulse off time decreased in pulsed mode experiments. PDEAEMA-based copolymer films were deposited to investigate their behavior under different pH conditions. The results indicate that the films exhibited distinct responses in acidic and basic environments.

## 1. Introduction

Stimuli-responsive polymers, also referred to as smart polymers, can alter their physical and/or chemical properties when exposed to external stimuli [1]. Due to their ability to reversibly respond to changes in environmental conditions, stimuli-responsive polymers find applications across a broad range of fields [2,3,4,5,6]. Various signals, such as electric/magnetic fields, pH, mechanical force, and temperature, can be employed to adjust the behavior of these intelligent polymers. pH-sensitive polymers respond to changes in environmental pH through the presence of ionizable groups [7]. This response involves a physical state change of the polymer, manifested as contraction or expansion due to the protonation or deprotonation of pendant groups of the polymer. Polymers containing pendant basic groups, such as amines and their derivatives, are referred to as polybases [8]. When the environmental pH falls below their pKa (the acid dissociation constant) value, these polymers become protonated and acquire a positive charge. As a result, electrostatic repulsion increases, and the polymer swells, exhibiting hydrophilic behavior. In contrast, when the pH rises above the pKa value, deprotonation occurs, causing the polymer chains to shrink and display hydrophobic characteristics [9,10]. Due to their stimuli-responsive behavior, these materials have significant applications in various fields, particularly in drug delivery [11,12,13,14,15].

Polymers can be synthesized using either solution-based [16,17,18,19] or vapor-based techniques [20,21,22]. Modern solution-based polymerization methods, such as Atom Transfer Radical Polymerization (ATRP) [23,24] and Reversible Addition–Fragmentation Chain Transfer (RAFT) [25], provide excellent control over polymer architecture; however, their reliance on organic solvents, long reaction times, and solvent-related impurities can damage delicate substrates and pose environmental and health concerns. In contrast, plasma polymerization, a vapor-based technique often implemented via Plasma-Enhanced Chemical Vapor Deposition (PECVD), utilizes energetic electrons to initiate polymerization, thereby eliminating solvent-related drawbacks and enabling a substrate-independent process. PECVD retains the advantages of conventional chemical vapor deposition, including being solvent-free, cost-effective, and environmentally friendly [26,27]. In addition, this method is time-efficient and can be applied to geometrically complex substrates [28,29]. In this process, high-energy electron collisions break the monomer bonds and create active species, i.e., radicals and ions. The recombination and rearrangement of these active species develop polymer films. Since collisions with electrons in plasma activate gases, almost all monomers can be processed by plasma polymerization. It is also a one-step and pinhole-free coating procedure [30]. In polymer synthesis via the PECVD technique, the most commonly used radio frequency (RF) plasmas are capacitively coupled plasma (CCP) and inductively coupled plasma (ICP) [31]. In a CCP system, the RF signal is applied between two electrodes, and the electric field generated between the electrodes ionizes the gas to form plasma. In an ICP system, an RF current is passed through a coil or inductor, generating a strong magnetic field in the gas via induction. In this configuration, energy is transferred to the gas through magnetic induction rather than an electric field [32]. While the electrodes in CCP are in direct contact with the plasma, the coils in ICP do not contact the reactive plasma gas [33,34]. Therefore, ICP provides more reliable plasma conditions. Moreover, plasmas generated in ICP are typically denser and more uniform than those in CCP [35,36,37], which favors the controlled deposition of polymer thin films. ICP-based plasma polymerization also overcomes key limitations of modern solution-based polymerization techniques, as discussed above, while enabling precise control over film growth directly on surfaces.

Poly(2-(diethylamino)ethyl methacrylate) (PDEAEMA) is a pH-responsive polymer with broad applications in drug and gene delivery [38,39,40,41]. This study aims to synthesize PDEAEMA thin films using the PECVD technique through an ICP system. Since plasma conditions influence the polymer film structure, the effects of plasma modes (continuous and pulsed), peak power, plasma pulse off time, and substrate temperature were examined. The chemical composition of the films was analyzed by X-ray Photoelectron Spectroscopy (XPS), while the chemical structure was characterized using Fourier Transform Infrared Spectroscopy (FTIR). Reactive species present in the plasma were identified through Optical Emission Spectroscopy (OES) measurements. Film thickness during deposition was monitored by laser interferometry, and thickness values were confirmed using a profilometer. Under various processing conditions, the highest deposition rate of the PDEAEMA polymer was determined. Additionally, the pH-responsive behavior of the deposited films was investigated.

## 2. Materials and Methods

### 2.1. Materials

**Figure 1:**
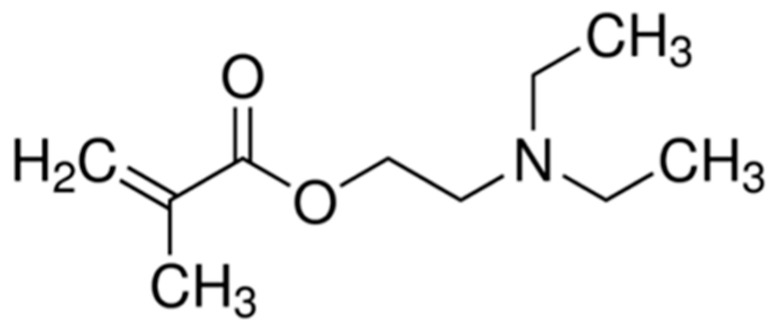
The chemical structure of the 2-(diethylamino)ethyl methacrylate (DEAEMA) monomer.

The monomer 2-(diethylamino)ethyl methacrylate (DEAEMA) (99%) was purchased from Sigma-Aldrich (Steinheim, Germany) and used as received without any further modification or purification. Flat silicon wafers (100, *p*-type) were used as substrates for polymer deposition. The structure of the DEAEMA monomer used in this study is given in Figure 1.

### 2.2. Plasma Polymerization

**Figure 2:**
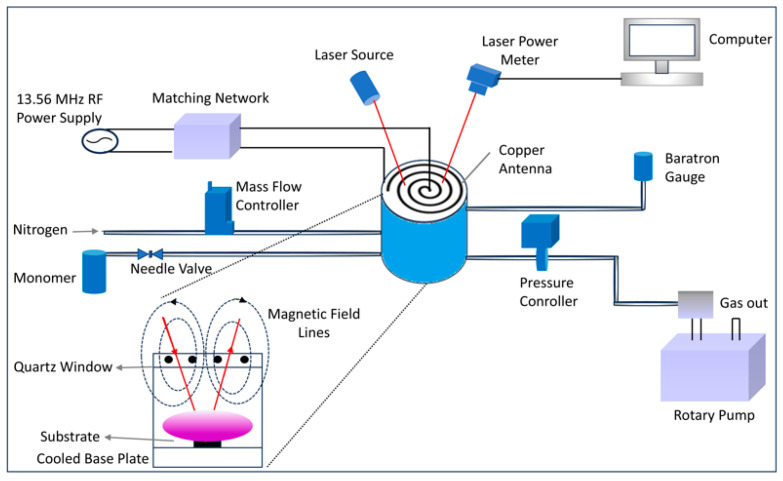
The schematic diagram of Inductively Coupled Plasma–Enhanced Chemical Vapor Deposition (ICP-PECVD)system. In the view showing the front part of the reactor, dashed lines represent the magnetic field lines, with arrows indicating their directions. The monomer vapor was supplied to the vacuum reactor at the desired flow rate using an on–off valve and a needle valve (Swagelok, Solon, OH, USA) installed on the line.

PDEAEMA films were deposited on flat silicon wafers via a PECVD system (Figure 2). The system is powered by a 13.56 MHz RF generator, and the RF power is inductively coupled to the plasma by a five-turn planar coil. The antenna had a 10 mm outer diameter and was placed above the 20 mm thick quartz window. An LC matching circuit was placed between the coil and the generator for impedance matching. The silicon wafers were placed in the middle of the water-circulated cooling stage. Before and after each experiment, the plasma chamber was purged with ultra-pure nitrogen gas flow. The system pressure was measured by a capacitance manometer, and the reactor was evacuated by a rotary pump at a base pressure of 20 mTorr. The reactor pressure was regulated using a butterfly throttling valve (MKS Instruments, Andover, MA, USA) installed between the pump and the reactor. To maintain a constant pressure at the desired setpoint during the experiments, a proportional–integral–derivative (PID) controller, connected to both the pressure gauge and the butterfly valve, was employed.

**Figure 3:**
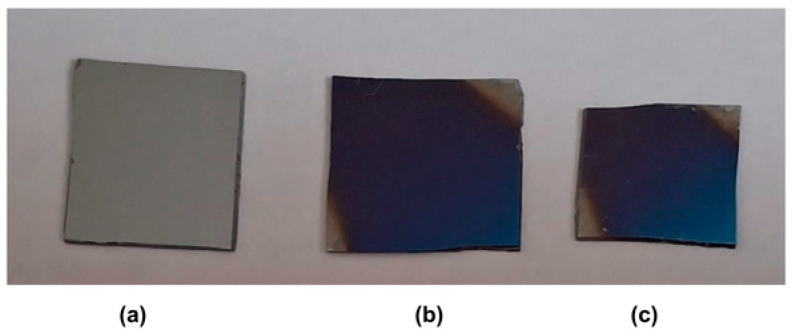
Photographs of silicon wafers showing (**a**) the uncoated substrate, (**b**) a film deposited in pulsed plasma mode (900 µs on-time, 100 µs off-time, 40 W peak power, 25 °C), and (**c**) a film deposited in continuous plasma mode (40 W, 25 °C).

The DEAEMA monomer was vaporized in a temperature-controlled stainless steel jar and delivered to the reactor through the metering valves. All deposition experiments were performed at 200 mTorr monomer pressure. To investigate the effect of pulsed discharge on plasma-polymer films, the depositions were carried out in both continuous and pulsed modes. The deposition parameters are summarized in Table 1. Representative photographs of the uncoated and plasma-polymer-coated substrates, prepared in both continuous and pulsed plasma modes, are shown in Figure 3.

### 2.3. PDEAEMA Film Characterizations

The thickness of the plasma-polymer films was monitored by laser interferometry, in which the reflectance of a 633 nm He–Ne laser beam (JDS Uniphase) from the silicon substrate was measured using a laser power meter. The thicknesses were also measured with ex situ methods (AEP 500LS profilometer, Santa Clara, CA, USA) in order to check the accuracy of the interferometric measurement results. The deposition rate was calculated from the ratio of film thickness to deposition time, and the deposition process was terminated upon reaching a film thickness of 100 nm. The chemical structure of the plasma-polymer films was analyzed using FTIR spectroscopy. Spectra were recorded with a Bruker Vertex 70 FTIR spectrometer (Bruker Optics GmbH, Ettlingen, Germany) over a wavenumber range of 400–4000 cm^−1^ at a resolution of 4 cm^−1^. All FTIR results were baseline corrected and normalized. The chemical structures of the polymer films were investigated with X-ray photoelectron spectroscopy (XPS, PHI 5000 VersaProbe, Physical Electronics Inc., Chanhassen, MN, USA) equipped with a monochromated Al Kα source. The wettability of the polymer films was measured using the sessile drop method with a Kruss Easy Drop contact angle goniometer (KRÜSS GmbH, Hamburg, Germany) system at ambient temperature. Pure water with a pH close to 7.0 at room temperature was used in the water contact angle measurements. Optical emission from the plasma was collected via an optical fiber positioned above the quartz window and transmitted to a spectrophotometer (Ocean Optics QE65000, Ocean Optics Inc., Dunedin, FL, USA)with a spectral range of 200–1100 nm. Detection was carried out using a CCD image sensor (Hamamatsu S7031-1006, Hamamatsu Photonics K.K., Hamamatsu, Japan) integrated into the spectrophotometer.

## 3. Results and Discussion

### 3.1. Deposition Rates

Film thickness was measured using laser interferometry as a real-time, non-contact technique. This method is based on the variation in laser beam intensity caused by the interference of light reflected from the front and back surfaces of the growing film during deposition. The phase difference between the reflections from the front and back surfaces is given by:

(1) $$\;\;\;\;\varphi =\frac{2\pi }{\lambda }(2n_{polymer}L)$$

where λ is the laser wavelength, $n_{polymer}$ is the refractive index of the film, and L is the film thickness. The film thickness change per interference fringe is given by:

(2) $$\frac{d}{fringe}=\frac{\lambda }{2n_{polymer}}$$

**Figure 4:**
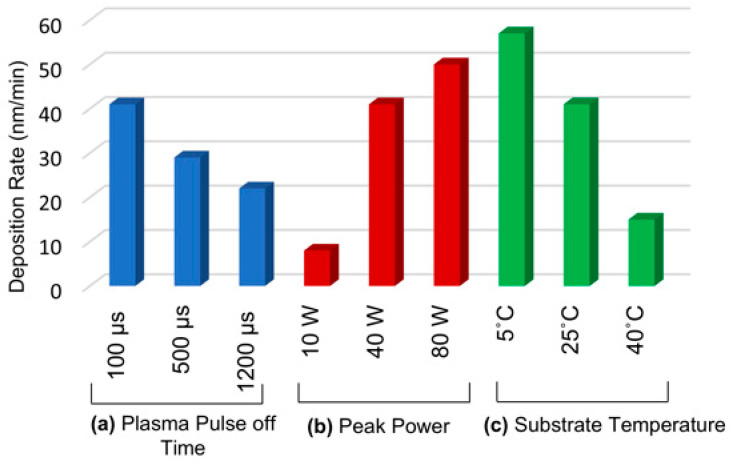
Deposition rates of the PDEAEMA polymer under different experimental conditions: (**a**) varying plasma pulse off time (blue bars) at a fixed plasma pulse on time of 900 µs, peak power of 40 W, and substrate temperature of 25 °C; (**b**) varying peak power (red bars) at a fixed plasma pulse on time of 900 µs, plasma pulse off time of 100 µs, and substrate temperature of 25 °C; (**c**) varying substrate temperature (green bars) at a fixed plasma pulse on time of 900 µs, plasma pulse off time of 100 µs, and peak power of 40 W.

The refractive index of the PDEAEMA polymer film is considered to be a constant value of 1.5. The *d*/fringe is approximately 211 nm for a 633 nm laser beam wavelength. Following the deposition process, the reliability of the interferometric thickness measurements was validated using ex situ profilometry, showing consistent results between the two methods. Figure 4 shows the influence of plasma peak power, plasma pulse off time, and substrate temperature on the PDEAEMA deposition rate. It was found that the deposition rate decreases as the substrate temperature increases. In CVD processes, the rate-limiting steps typically involve adsorption (mass transfer) and the kinetics of surface reactions [42]. The high deposition rate at low substrate temperature is attributed to the dominance of the adsorption mechanism over surface reaction kinetics in the PDEAEMA polymer coatings produced in this study. Specifically, at low substrate temperature, the temperature difference between the plasma medium and the surface promotes faster and more extensive adsorption of reactive species onto the surface. Despite the high deposition rate, the chemical species adsorbed on the surface differed from the desired polymer structure, as revealed by FTIR analysis.

In experiments investigating the influence of plasma power on deposition rate, it was found that the rate of deposition increased with rising plasma power. This trend can be explained by the Yasuda parameter *W/FM* [43], where *W* represents the applied plasma power and *FM* denotes the mass flow rate of the monomer. With increasing plasma power, the energy supplied per molecule rises at a constant flow rate.

Consequently, high levels of monomer fragmentation occur in the plasma environment, and some molecules may even dissociate into atoms, resulting in faster surface coverage by reactive species. At high plasma power, the distortions observed in the FTIR peaks around 1200 cm^−1^ confirm the extensive fragmentation occurring in the plasma. Increasing the plasma pulse off time resulted in a decrease in the deposition rate. This can be attributed to a reduction in the average applied power. As previously mentioned, increasing the plasma power can increase the deposition rate. When the plasma pulse off time is long compared to the monomer’s residence time in the reactor, the plasma primarily interacts with fresh monomer. Conversely, if the plasma pulse off time is short relative to the monomer’s residence time, previously decomposed monomers remain in the plasma environment, leading to increased fragmentation when the plasma is reactivated.

### 3.2. Film Characterizations

**Figure 5:**
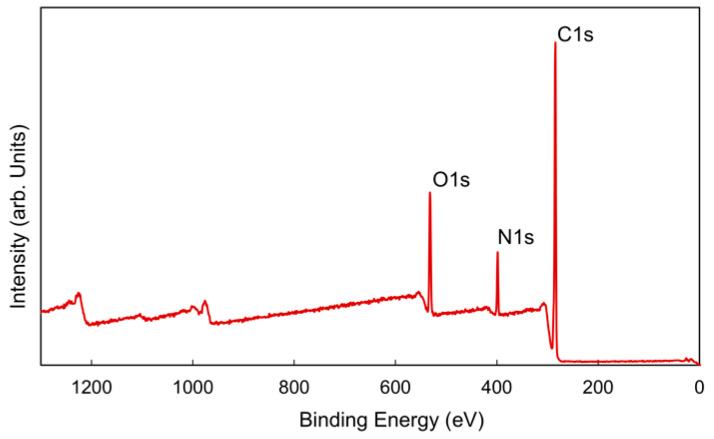
X-ray Photoelectron Spectroscopy (XPS) survey spectra of the PDEAEMA polymer deposited at a plasma pulse on time of 900 µs, plasma pulse off time of 100 µs, peak power of 40 W, and substrate temperature of 25 °C.

**Figure 6:**
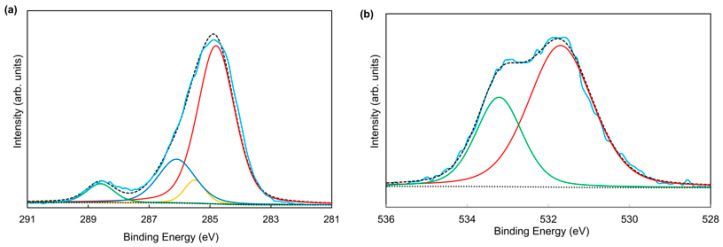
(**a**) Curve-fitted high-resolution C1s XPS spectrum of the PDEAEMA thin film. Red = C–H, yellow = C–C, dark blue = C–N, and green = C=O. (**b**) Curve-fitted high-resolution O1s XPS spectrum of the PDEAEMA thin film. Green = O–C and red = C=O The raw spectra are shown in light blue, and the corresponding curve-fitting results are indicated by dashed lines.

The chemical structure of PDEAEMA was verified using XPS analysis. The survey spectrum, which displays peaks corresponding to carbon, oxygen, and nitrogen at their characteristic binding energies, is presented in Figure 5. The atomic concentrations of carbon, oxygen, and nitrogen were determined to be 78%, 14%, and 8%, respectively. The C:O:N atomic ratio was calculated to be 9.8:1.8:1, which closely matches the theoretical DEAEMA ratio of 10:2:1. To investigate the bonding states of PDEAEMA, high-resolution C1s and O1s scans were obtained. The structure of PDEAEMA contains tertiary amine groups, and the N1s spectrum can be curve-fitted with only one peak component at a binding energy (BE) of 398.4 eV [44]. Figure 6a,b shows the high-resolution C1s and O1s regions of the spectra.

In previous studies, the C1s and O1s spectra of similar polymers were fitted with four and two subpeaks, respectively [45]. In the present study, the C1s subpeaks were C–H (~284.7 eV), C-C (~285.5 eV), C-N (~286.1 eV), and C=O (~288.6 eV), while the O1s subpeaks were C=O (~531.8 eV) and O-C (~533.1 eV). These results suggest that the chemical bonds identified in the high-resolution XPS spectra verify the successful synthesis of the PDEAEMA polymer

**Figure 7:**
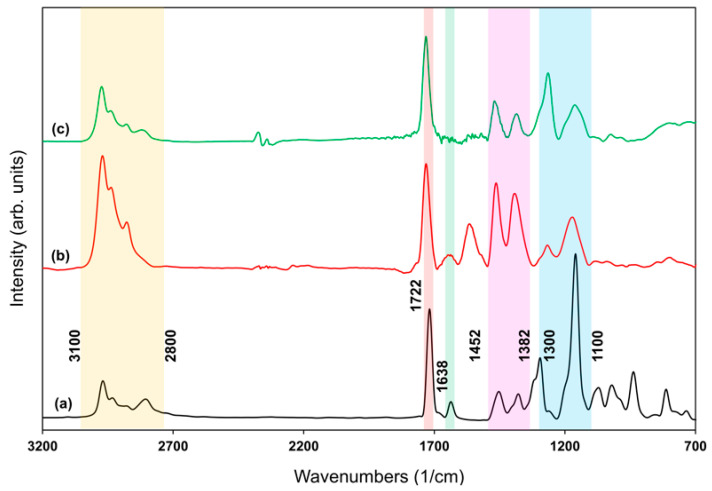
Fourier Transform Spectroscopy (FTIR) spectra of the (**a**) DEAEMA monomer (black curve), (**b**) PDEAEMA deposited in continuous plasma mode (40 W plasma power, substrate temperature 25 °C; red curve), and (**c**) PDEAEMA deposited in pulsed plasma mode (plasma pulse on time 900 µs, plasma pulse off time 100 µs, peak power 40 W, substrate temperature 25 °C; green curve). Highlighted regions indicate selected spectral regions for visual guidance.

In addition to the XPS analysis, the chemical structure of the polymeric films was further examined by FTIR spectroscopy. Figure 7 shows the FTIR spectra of the DEAEMA monomer and the spectra of PDEAEMA deposited at continuous and pulsed modes. All spectra were normalized for thickness and corrected for baseline. The characteristic peaks in the monomer spectrum were C-H stretching (3100–2800 cm^−1^), C=O stretch at 1722 cm^−1^, C=C bond at 1638 cm^−1^, C-H bending (1452–1382 cm^−1^), C-O/C-N stretching at 1294 cm^−1^, and C-O-C stretching at 1160 cm^−1^. The peaks at 2971, 2934, 2809 cm^−1^ correspond to the stretching vibration of C-H due to the –N(C_2_H_5_)_2_ group [45,46]. To quantitatively evaluate the relative functional group retention and changes in the functional group composition of the plasma-polymerized films, the FTIR-derived peak intensity ratios and FWHM values are summarized in Table 2. Due to overlapping stretching vibrations, the absorption band centered at 2971 cm^−1^ was treated as a single peak for quantitative analysis, and the bands at 2971 cm^−1^, 1294 cm^−1^, and 1160 cm^−1^ were selected as representative of characteristic functional groups of the monomer structure sensitive to structural changes under different plasma conditions. The intensities of these peaks were normalized to the carbonyl (C=O) stretching peak at 1722 cm^−1^, which was selected as an internal reference due to its sharp and well-defined profile across all samples.

As shown in Figure 7, the vibration peak observed between 1614 cm^−1^ and 1496 cm^−1^ in the continuous mode polymer spectrum may correspond to undesired structural features, which was absent in both the monomer and the pulsed polymer spectra. In continuous mode, film deposition and bombardment of the polymer surface by high-energy plasma species occur simultaneously, leading to surface damage and deterioration of the polymer’s structural integrity. The absence of a peak at 1638 cm^−1^, corresponding to the C=C bond, in the pulsed plasma polymer indicates that polymerization proceeds through the acrylate double bond [47]. In contrast, the presence of this peak in the continuous mode film suggests the existence of residual monomeric species. Pulsing the discharge minimizes undesirable monomer fragmentation and promotes the formation of a more uniform and well-defined polymer structure [48].

This behavior is attributed to the deposition mechanism in pulsed plasma, where monomer fragmentation occurs predominantly during the plasma on period, while polymer chain growth proceeds during the plasma off period, thereby reducing the incorporation of unreacted monomer into the film [49]. A detailed examination of the peak parameters highlights differences in polymerization behavior between continuous and pulsed plasma deposition modes. Both films exhibited broadening of the 1160 cm^−1^ band, reflecting plasma-induced structural modifications. However, the peak intensity ratios for the pulsed plasma film were generally closer to those of the monomer. Overall, the pulsed polymer had a closer resemblance to that of the monomer, indicating a more complete polymerization.

**Figure 8:**
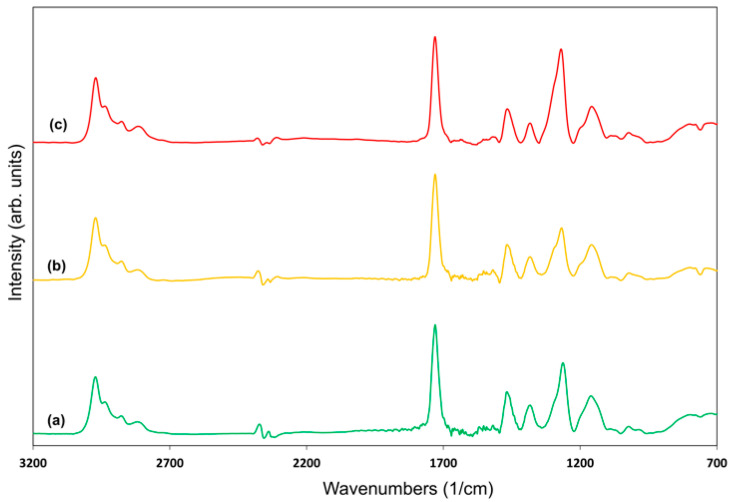
FTIR spectra of polymers deposited in pulsed plasma mode: (**a**) plasma pulse off time 100 µs (green curve), (**b**) plasma pulse off time 500 µs (yellow curve), and (**c**) plasma pulse off time 1200 µs (red curve), at a plasma pulse on time of 900 µs, peak power 40 W, and substrate temperature 25 °C.

No significant differences were observed in the spectra of the polymer films deposited at different plasma pulse off times (Figure 8). The average power supplied to the plasma was calculated by multiplying the peak plasma power by the duty cycle, which is defined as the ratio of the plasma on time to the total pulse period. Increasing the plasma off time reduces the duty cycle and the average power, leading to reduced monomer fragmentation. As a result, the incorporation of undesired groups into the polymer structure is minimized.

**Figure 9:**
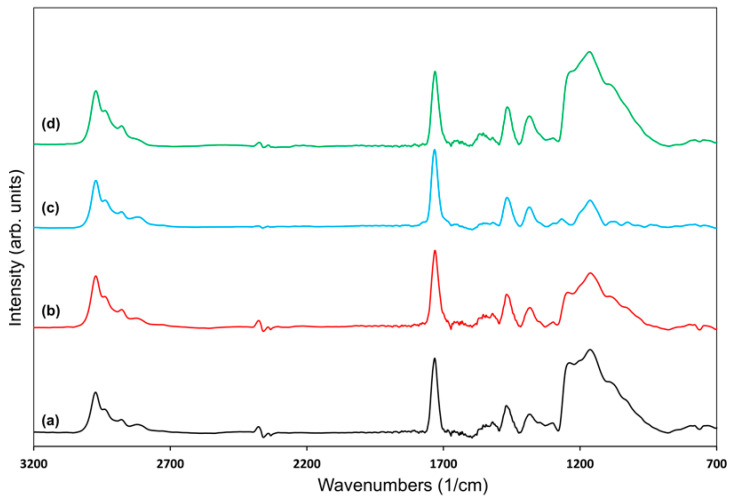
FTIR spectra of polymers deposited under different conditions: (**a**,**b**) substrate temperatures of 5 °C (black curve) and 40 °C (red curve) at a plasma pulse on time of 900 µs, plasma pulse off time of 100 µs, and peak power of 40 W; (**c**,**d**) peak powers of 10 W (blue curve) and 80 W (green curve) at a plasma pulse on time of 900 µs, plasma pulse off time of 100 µs, and substrate temperature of 25 °C.

The effect of plasma peak power and substrate temperature on the deposited polymer film was also investigated (Figure 9). For the films deposited at 5 °C, 40 °C substrate temperature, and 80 W peak power, the band at 1294 cm^−1^ showed strong overlap with the dominant band at 1160 cm^−1^, appearing as a shoulder. Therefore, the peak intensity ratios were not calculated for the 1294 cm^−1^ band to avoid overestimation or misinterpretation. In contrast, the peak intensity ratio *I_1160_/I_1722_* was evaluated, and the structural variations in this spectral region were further assessed using FWHM_1160_. As the plasma power increased, the deposited film structure deviated from that of the polymer, particularly in the 1300–1100 cm^−1^ region. As mentioned previously, according to the Yasuda parameter, energetic monomer molecules at high plasma power can lead to fragmentation and the formation of new radical species, which may disrupt the polymer structure. High-energy species can recombine randomly, thereby altering the polymer structure [50]. This trend is reflected in the FTIR analyses (Table 2). At a high plasma power of 80 W, a significant increase in the FWHM of the 1160 cm^−1^ was observed. This indicates that higher power leads to greater structural broadening and deviation from the monomer, consistent with extensive fragmentation. Comparing the plasma powers of 10 W and 40 W, both the FWHM of the 1160 cm^−1^ peak and the intensity ratios showed that the values at 40 W were closer to those of the monomer. This indicates that 40 W plasma power provides a polymer structure that best preserves the monomer’s functional groups through controlled polymerization.

Regarding the effect of substrate temperature on the structural characteristics of the films, the FTIR spectra revealed clear trends in the FWHM of the 1160 cm^−1^ peak (Table 2). Both low (5 °C) and high (40 °C) substrate temperatures resulted in increased FWHM values, indicating structural disorder. At lower substrate temperatures (5 °C), the surface mobility of adsorbed species was significantly reduced, which limits their capacity to reorganize into energetically favorable and well-defined configurations. This restricted rearrangement leads to a higher degree of structural disorder and the incorporation of undesired functional groups, as reflected in the broadening of the peak around 1200 cm^−1^. At higher substrate temperatures, enhanced monomer fragmentation due to increased gas-phase reactions may occur, leading to the incorporation of additional undesired chemical groups on the polymer surface. In contrast, the film deposited at 25 °C exhibited a narrower FWHM and a lower *I_1160_/I_1722_* ratio, suggesting more controlled polymerization and a more ordered polymer structure.

**Figure 10:**
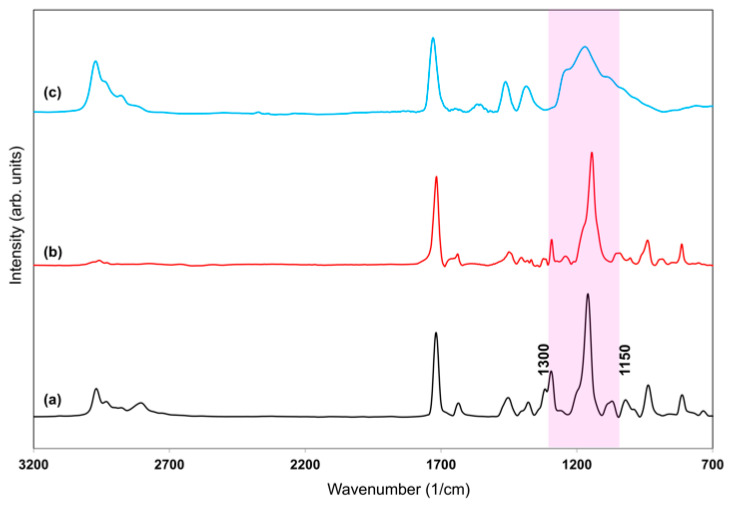
FTIR spectra of (**a**) DEAEMA monomer (black curve), (**b**) ethylene glycol dimethacrylate (EGDMA) monomer (red curve), and (**c**) the cross-linked P(DEAEMA-EGDMA) copolymer film (blue curve) deposited at a plasma pulse on time of 900 µs, plasma pulse off time of 100 µs, peak power of 40 W, and substrate temperature of 25 °C. Highlighted region indicates the C–O stretching vibrations for all spectra.

To prevent dissolution of the PDEAEMA film during the pH responsivity experiments, ethylene glycol dimethacrylate (EGDMA) was co-introduced into the reactor together with DEAEMA. EGDMA acts as a crosslinking agent, linking polymer chains through covalent bonds to form a three-dimensional network structure, thereby improving the water resistance of the films. The simultaneous introduction of DEAEMA and EGDMA monomers into the reactor led to the formation of a crosslinked P(DEAEMA-EGDMA) copolymer film. FTIR spectra of DEAEMA, EGDMA, and P(DEAEMA-EGDMA) are given in Figure 10. The FTIR spectrum of EGDMA showed strong similarity to that of the DEAEMA monomer. The peaks observed between 1300–1150 cm^−1^ in the DEAEMA monomer spectrum, which correspond to the C–O stretching vibrations, overlapped with those of the EGDMA in the same range, leading to spectral broadening in the copolymer film. In addition, the C–H stretching vibrations associated with the –N(C_2_H_5_)_2_ group of DEAEMA, are also present at 2971, 2934, and 2809 cm^−1^ in the copolymer spectrum. The presence of characteristic peaks from both monomers in the copolymer spectrum, together with the observed spectral broadening, confirms the successful deposition of P(DEAEMA-EGDMA) copolymer film.

### 3.3. Plasma Characterizations

**Figure 11:**
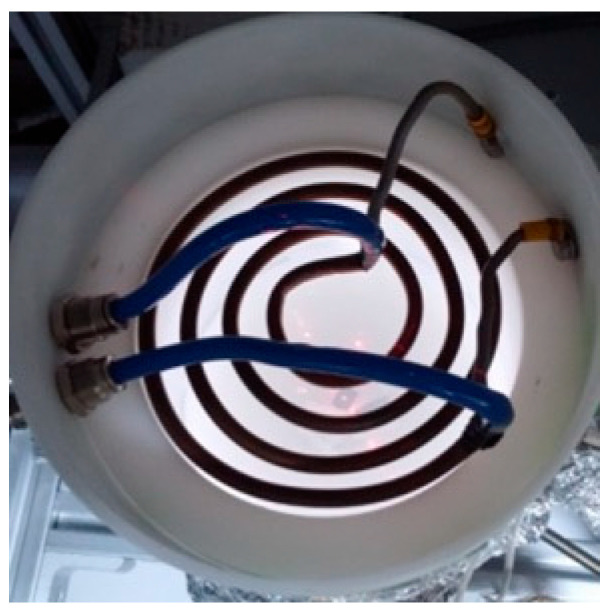
Top-view image of the plasma reactor during deposition, showing the plasma glow of the DEAEMA monomer.

**Figure 12:**
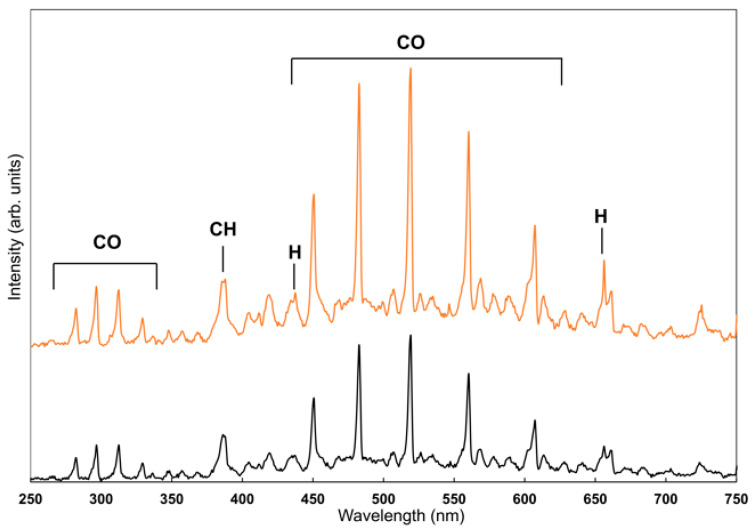
Optical Emission Spectroscopy (OES) spectra of the plasma at peak powers of 40 W (black curve) and 80 W (orange curve) with a plasma pulse on time of 900 µs and pulse off time of 100 µs.

OES is a powerful diagnostic technique widely used to characterize the chemical composition and species densities within plasmas. Detailed information about the plasma environment can be obtained by analyzing the wavelength and intensity distribution of the OES spectra, which arise from the radiation emitted as excited atoms and molecules relax from higher to lower energy states [51]. A top view of the ICP reactor during deposition, exhibiting the characteristic plasma glow of the DEAEMA monomer, is shown in Figure 11. OES spectra of the DEAEMA monomer plasmas generated at peak powers of 40 W and 80 W were compared. The comparative spectra are presented in Figure 12. Spectral peak assignments were performed using reference data from the literature [52,53]. The OES analysis revealed the presence of CO, CH, C_3_, C_2_, H_2_, and H species in the DEAEMA monomer plasma. The lines belonging to CO transitions were observed in the 283–371 nm and 451–664 nm regions, and the distinct peak at 388.6 nm corresponded to CH transitions. The emission spectra of the H_2_ molecule were in the 570–650 nm region. The low-intensity peaks at 404.1 nm and 468.5 nm refer to the C_3_ and C_2_ species, respectively. Only hydrogen lines were clearly visible among atomic emissions, especially at 435.1 nm and 656.2 nm. Additionally, the peaks at 357 nm and 368.4 nm may correspond to nitrogen-containing fragments, since nitrogen structures give emission spectra in this region [54]. All of these species contribute to the plasma polymerization process. According to the spectral analysis, the most prominent species in the plasma were CO and CH. These species play a critical role in forming reactive fragments and promoting the growth of plasma-polymer films. Raising the applied power from 40 W to 80 W led to a corresponding increase in the peak intensities. The hydrogen atomic lines at 435.1 nm and 656.2 nm showed a sharp increase at 80 W, indicating enhanced monomer fragmentation under higher power conditions. This result is consistent with both the increase in deposition rate and the structural degradation, as seen in the FTIR spectra at 80 W. High-energy hydrogen species may also induce surface damage upon impact, leading to structural disorder within the polymer film.

### 3.4. pH Responsivity Analysis

**Figure 13:**
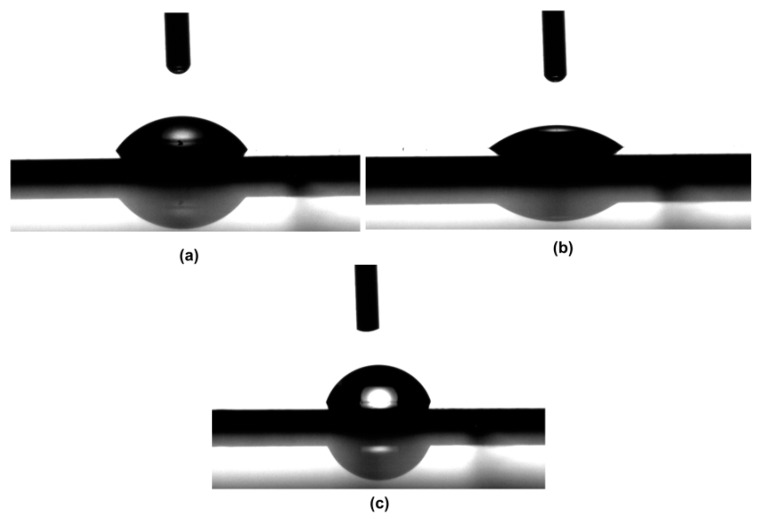
Contact angle images of the P(DEAEMA-EGDMA) copolymer film copolymer film before treatment (**a**), after exposure to (**b**) acidic solution and (**c**) basic solution. The corresponding average water contact angle values were 56.4 ± 1.2°, 36.9 ± 1.3° and 76.7 ± 1.7°, respectively.

**Figure 14:**
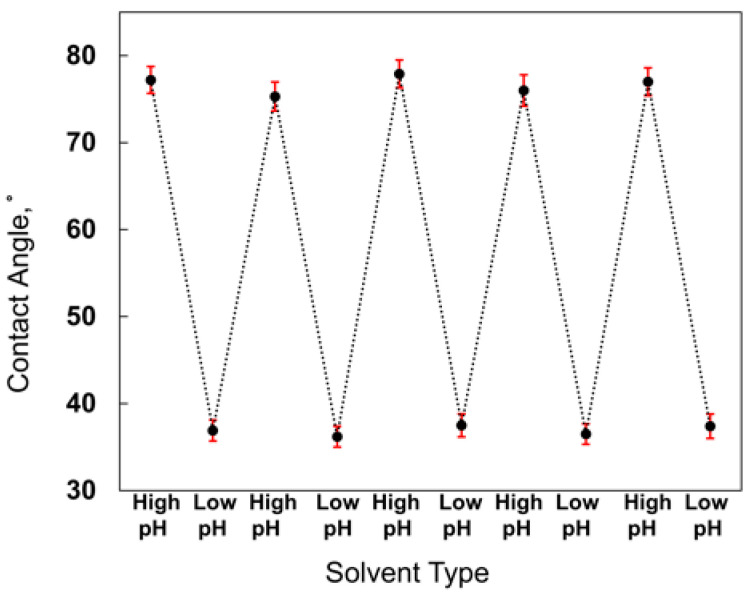
Reversibility and stability of contact angle measurements on P(DEAEMA-EGDMA) coated surfaces over five consecutive cycles of acidic (pH 2.75) and basic (pH 8.5) treatments. Data points represent the mean values, and error bars denote the respective standard deviations for each individual cycle (*n* = 3 for each point).

The pH-responsivity of the plasma-polymer films was examined by treating the polymer surfaces with acidic and basic solutions. The water contact angle of the untreated polymer surface was measured as 56.4 ± 1.2°, serving as a reference for the fully non-ionized state. The surface of the copolymer film was treated by immersing it in acidic (HCl solution with pH 2.75) and basic (NaOH solution with pH 8.5) solutions. Figure 13 shows the water droplets on the acid and base-treated surfaces. After the acidic and basic treatments, the film was rinsed with water and dried with pure nitrogen. The surfaces were exposed to the solutions for 10 s. The water contact angle was measured after every treatment and measured as 36.9 ± 1.3° and 76.7 ± 1.7° for acidic and basic treatments, respectively. The reversible response of the surface was confirmed over multiple cycles (Figure 14); the minimal variations in the error bars (with standard deviations ranging from 1.2° to 1.9°), the statistical reproducibility, and the consistent surface behavior of the coating. The difference in contact angle values can be attributed to the basic polymer behavior of PDEAEMA.

It is established that polymers with basic monomers become cationic under acidic conditions due to protonation of the basic groups, whereas polymers with acidic monomers become anionic under basic conditions as a result of deprotonation [8]. The pKa of PDEAEMA is reported to be around 7 [55], indicating that protonation and deprotonation of the polymer chains occur near neutral pH values. When the ambient pH is lower than the pKa, the tertiary amine groups of PDEAEMA are protonated, introducing positive charges along the polymer backbone.

These charges generate electrostatic repulsion between the polymer chains, causing them to expand and exposing hydrophilic groups to the surface, which facilitates water spreading and reduces the contact angle. Conversely, when the ambient pH is above the pKa of PDEAEMA, the amine groups are deprotonated, and the polymer chains lose their positive charges. In this state, hydrophobic interactions between polymer segments become dominant, leading to chain contraction and aggregation, which results in a more hydrophobic surface and an increased contact angle. In the present study, a significant fraction of the polymer was expected to remain positively charged at pH 8.5; however, the degree of deprotonation was sufficient to induce a clear hydrophobic transition. This is evidenced by the substantial increase in the contact angle to 76.7 ± 1.7°, which was higher than both the untreated surface (56.4 ± 1.2°) and the acidic-treated surface (36.9 ± 1.3° at pH 2.75). The fact that the untreated value fell between the acidic and basic values further confirmed the pH-responsive behavior of the polymer.

## 4. Conclusions

In the present study, pH-responsive thin films based on PDEAEMA were successfully synthesized via a planar ICP-PECVD system, utilizing both continuous and pulsed operation modes. FTIR analysis confirmed the preservation of characteristic functional groups. However, the pulsed plasma films exhibited a closer resemblance to the monomer structure compared to the continuous mode films, with reduced incorporation of residual monomeric species. The highest deposition rate among the investigated conditions was obtained at a substrate temperature of 5 °C, while the polymer film exhibited the most desirable structure at 25 °C. Moreover, regarding the plasma pulse off time, all tested off-time conditions maintained the functional groups in the polymer. For peak power, the polymer structure was degraded at the highest applied power of 80 W, while the optimal polymer structure was achieved at 40 W. OES provided insight into the plasma environment by revealing reactive chemical species and demonstrated that plasma power significantly influenced the density of reactive species during deposition. The pH-responsive behavior of the films was evidenced by reversible changes in the water contact angle. Acidic treatment increased the surface hydrophilicity due to the protonation of tertiary amine groups below their pKa, whereas basic treatment led to a more hydrophobic surface. These results demonstrate that the ICP-PECVD process enables the fabrication of stable, functional thin films suitable for smart coating and sensing applications. While this study qualitatively demonstrated responsiveness through contact angle measurements, future work will focus on quantitative swelling characterization, such as thickness changes and swelling ratios, to further enhance the application-oriented evaluation of these plasma-polymerized smart coatings.

## Figures and Tables

**Table 1:** PECVD parameters for poly(2-(diethylamino)ethyl methacrylate) (PDEAEMA) deposition.

Plasma Mode	Plasma Pulse on Time (µs)	Plasma Pulse off Time (µs)	Power (W)	Peak Power (W)	Substrate Temperature (°C)
Continuous	N/A	N/A	40	N/A	25
Pulsed	900	100	N/A	40	25
Pulsed	900	500	N/A	40	25
Pulsed	900	1200	N/A	40	25
Pulsed	900	100	N/A	40	5
Pulsed	900	100	N/A	40	40
Pulsed	900	100	N/A	10	25
Pulsed	900	100	N/A	80	25

Note: N/A, Not applicable.

**Table 2:** Quantitative FTIR analysis of the DEAEMA monomer and plasma-polymerized PDEAEMA films.

Sample (Deposition Mode)	Varied Parameter	*I_2971_/* *I_1722_*	*I_1160_/* *I_1722_*	FWHM_1160_ (cm^−1^)	*I_1294_/* *I_1722_*
Monomer	Reference	0.34	1.50	30.86	0.55
Mode effect					
PP–Continuous	25 °C, 40 W	1.07	0.49	63.64	0.70
PP–Pulsed	25 °C, 40 W	0.52	0.35	65.58	0.62
Pulse off time effect					
PP–Pulsed	100 μs off time	0.52	0.35	65.58	0.62
PP–Pulsed	500 μs off time	0.60	0.34	59.80	0.50
PP–Pulsed	1200 μs off time	0.61	0.34	61.72	0.89
Subs. temp. effect					
PP–Pulsed	5 °C	0.55	1.13	200.57	overlapped
PP–Pulsed	25 °C	0.52	0.35	65.58	0.62
PP–Pulsed	40 °C	0.66	0.70	187.07	overlapped
Peak power effect					
PP–Pulsed	10 W	0.60	0.34	75.2	0.11
PP–Pulsed	40 W	0.52	0.35	65.58	0.62
PP–Pulsed	80 W	0.72	1.27	200.57	overlapped

Note: PP denotes plasma-polymerized PDEAEMA films. For continuous mode, the applied power corresponds to the average power, whereas for pulsed mode, the reported power represents the peak power. $I_{x}/I_{y}\;$ represents the intensity ratio of the absorption bands at x and y cm^−1^. All intensity ratios are given relative to the carbonyl band at 1722 cm^−1^. FWHM_1160_ indicates the full width at half maximum of the 1160 cm^−1^ band, reflecting structural disorder. “Overlapped” indicates that the intensity ratio calculation was not performed due to peak merging under those specific conditions. Bold text indicates main headings and subgroup headings within the table.

## Data Availability

The original contributions presented in this study are included in the article. Further inquiries can be directed to the corresponding author.
